# Supplementary material for: Study of the Influence of Technological Parameters on Generating Flat Part with Cylindrical Features in 3D Printing with Resin Cured by Optical Processing
Source: Polymers (Basel). 2020 Aug 27;12(9):1941. doi: 10.3390/polym12091941 (PMC7564599; doi:10.3390/polym12091941)
Supplement: Supplementary file 1 [file polymers-12-01941-s001.pdf]

polymers

Supplementary Materials

Study of the influence of technological parameters on generating flat part with cylindrical features in 3D printing with resin cured by optical processing

Aurel Tulcan [1,*], Mircea Dorin Vasilescu [2,*] and Liliana Tulcan [2,*]

[1] Department of IMF, Politehnica University Timisoara; aurel.tulcan@upt.ro,
[2] Department of MMUT, Politehnica University Timisoara; mircea.vasilescu@upt.ro
* Correspondence: aurel.tulcan@upt.ro (A.T.); mircea.vasilescu@upt.ro (M.D.V.); liliana.tulcan@upt.ro (L.T.)

Received: 22 July 2020; Accepted: 24 August 2020; Published: date

1. The part probing strategy.

Table S1. Definition of the part probing strategy.

Physical Feature	Measured feature	Z-coordinate of the measuring plane MP in mm	N° of contact points
Surface A	Plane	0	110
Surface B	Straight Line	-2	14
Surface C	Straight Line	-2	12
Surface B1	Straight Line	-2	14
Surface C1	Straight Line	-2	12
Contour X1	Straight Line	0	12
Contour X2	Straight Line	0	12
Contour Y1	Straight Line	0	20
Contour Y2	Straight Line	0	20
Hole Φ 5	Circle	-2.5	36
Hole Φ 10	Circle	-2.5	72
Hole Φ 15	Circle	-2.5	72
Cylinder Φ 5	Circle MP 1 / Circle MP 2 / Circle MP 3	2 / 5 / 8	36 / 36 / 36
Cylinder Φ 10	Circle MP 1 / Circle MP 2 / Circle MP 3	2 / 5 / 8	72 / 72 / 72
Cylinder Φ 15	Circle MP 1 / Circle MP 2 / Circle MP 3	2 / 5 / 8	72 / 72 / 72

2. The experimental value for supports.

Table S2. The experimental value for supports of the feature generated by 3D printing from solid design 3D element.

N°	Type	The diameter of the contact surface in mm	Support density in %	Number of generated supports	Number of broken supports	Flatness in mm
	(1)	(2)	(3)	(4)	(5)	(6)
1	Light	*0,8*	*30*	***243***	***243***	*Not possible*
2		0,8	40	418	154	0,853
3		0,8	50	868	95	0,830
4		0,8	60	927	45	0,823
5	Medium	*1,2*	*30*	*92*	*34*	*1,031*
6		1,2	40	180	15	0,649
7		1,2	50	296	1	0,584
8		1,2	60	421	0	0,763
9	Heavy	*1,6*	*30*	*56*	*16*	*0,646*
10		1,6	40	89	2	0,631
11		1,6	50	158	0	0,495
12		1,6	60	229	0	0,700

3. The Standardized Pareto Chart and Estimated Response Surface for Broken supports and Flatness.

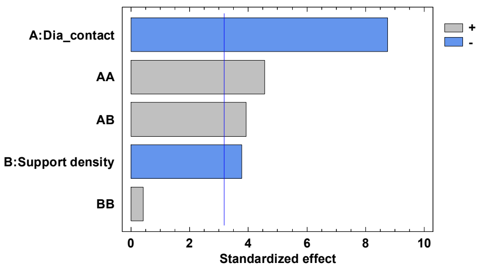

Figure S1. Standardized Pareto Chart for Broken supports.

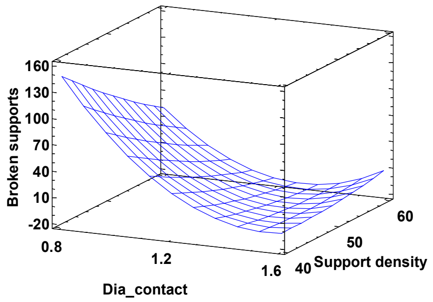

Figure S2. Estimated Response Surface for broken supports.

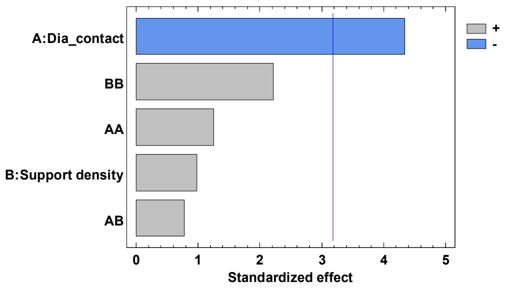

Figure S3. Standardized Pareto Chart for Flatness.

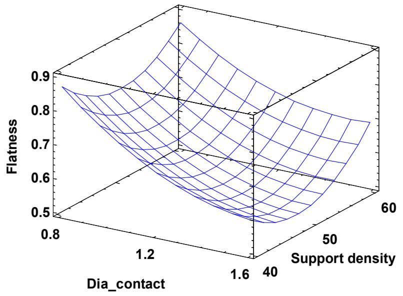

Figure S4. Estimated Response Surface for Flatness.

4. The experimental value for dimension supports.

Table S3. The experimental value for dimension supports of the feature generated by 3D printing from solid design 3D element.

	Independent factors			Number of supports		Response variable
Run	Support density in %	Contact depth in mm	The diameter of the contact surface in mm	Total	Broken	
1	40	0,1	1,2	190	33	
2	40	0,2	1,6	112	1	
3	40	0,3	1,2	214	11	
4	40	0,2	0,8	484	7	
5	50	0,2	1,2	296	1	
6	50	0,1	1,6	193	1	
7	50	0,1	0,8	868	95	
8	50	0,3	0,8	909	4	
9	50	0,2	1,2	296	1	
10	50	0,2	1,2	296	0	
11	50	0,3	1,6	158	0	
12	60	0,3	1,2	466	2	
13	60	0,2	1,6	269	0	
14	60	0,2	0,8	982	4	
15	60	0,1	1,2	479	1	

5. The dimensional value for correction the nominal dimension of the part body.

Table S4. Dimensional value for correction the nominal dimension of the part body printed elements.

No	Dimension type	Nominal value in mm	Measured value in mm	Extension/ Contraction value in mm	Printed value in mm	Obs.
1	Cylindrical elements	5	5,24	0,24	4,76	
2		10	10,46	0,46	9,54	
3		15	15,68	0,68	14,32	
4	Cylindrical hole elements	5	5,09	0,09	4,91	
5		10	10,18	0,18	9,82	
6		15	15,42	0,42	14,58	
7	Linear elements	55	57,54	2,54	52,46	
8		35	37,04	2,04	32,96	
9		5	4,85	-0,15	5,15	
10	Hole Y center point dimension	5	5,29	0,29	4,71	
11		8	8,47	0,47	7,53	
12		15	15,87	0,87	14,13	
13	X center point dimension for hole	15	15,69	0,69	14,31	
14		5	5,23	0,23	4,77	
15		8	8,37	0,37	7,63	
16	Y center point dimension for cylinder	5	5,29	0,29	4,71	
17		8	8,47	0,47	7,53	
18		12	12,7	0,7	11,30	
19	X center point dimension for cylinder	25	26,15	1,15	23,85	
20		5	5,23	0,23	4,77	
21		7	7,32	0,32	6,68	

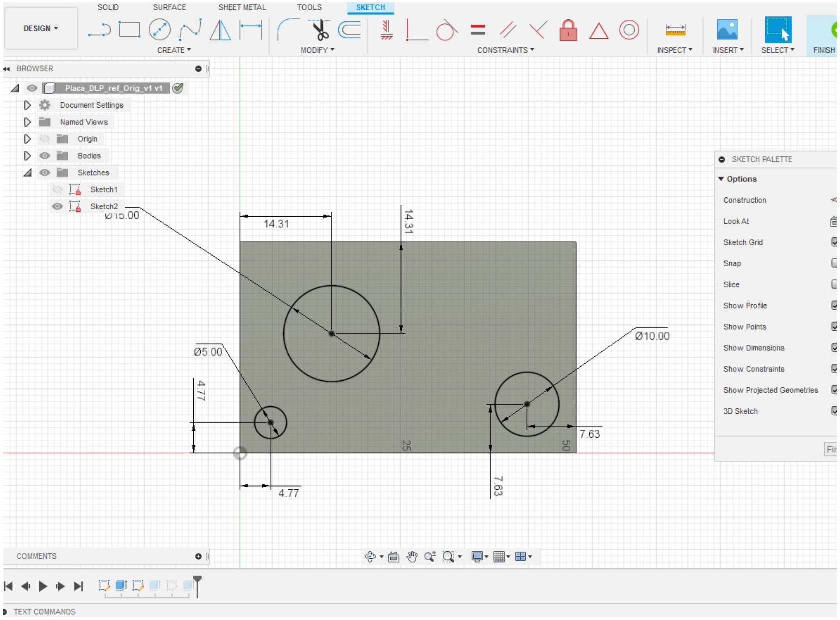

Figure S5. Center point dimension for hole generated in sketch 2.

6. The experimental value for Flatness.

Table S5. The experimental value for Flatness.

	Independent factors			Response variable
**Run**	**Support density in %**	**Contact depth in mm**	**The diameter of the contact surface in mm**	**Flatness in mm**
1	40	0,1	1,2	1,209
2	40	0,2	1,6	1,011
3	40	0,3	1,2	1,015
4	40	0,2	0,8	1,083
5	50	0,2	1,2	0,584
6	50	0,1	1,6	0,865
7	50	0,1	0,8	0,830
8	50	0,3	0,8	1,032
9	50	0,2	1,2	0,583
10	50	0,2	1,2	0,586
11	***50***	***0,3***	***1,6***	***0,495***
12	60	0,3	1,2	0,865
13	60	0,2	1,6	0,890
14	60	0,2	0,8	0,907
15	60	0,1	1,2	0,925

7. The Analysis of Variance for Flatness.

Table S6. Analysis of Variance for Flatness.

Source	*Sum of Squares*	*Df*	*Mean Square*	*F-Ratio*	*P-Value*
A: Support density	0.0667951	1	0.0667951	13.58	***0.0142***
B: Contact depth	0.0222605	1	0.0222605	4.53	0.0867
C: Dia.contact	0.0436601	1	0.0436601	8.88	***0.0308***
AA: Square Support density	0.317793	1	0.317793	64.61	***0.0005***
AB: Interaction Support density with Contact depth	0.004489	1	0.004489	0.91	0.3833
AC: Interaction Support density with Dia.contact	0.00075625	1	0.00075625	0.15	0.7111
BB: Square Contact depth	0.0587354	1	0.0587354	11.94	***0.0181***
BC: Interaction Contact depth with Dia.contact	0.081796	1	0.081796	16.63	***0.0096***
CC: Square Dia.contact	0.0335867	1	0.0335867	6.83	***0.0475***
Total error	0.0245918	5	0.00491835		
Total (corr.)	0.617948	14			

68 **8. The Estimated Response Surface for Flatness, Straightness_X1 and Straightness_X2.**

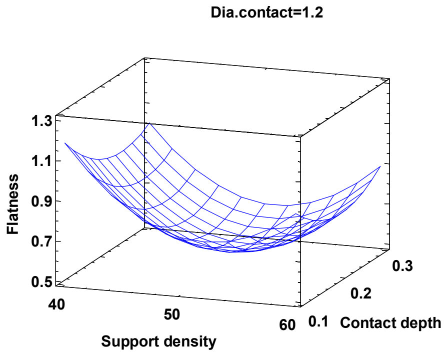

Figure S6. Estimated Response Surface for Flatness.

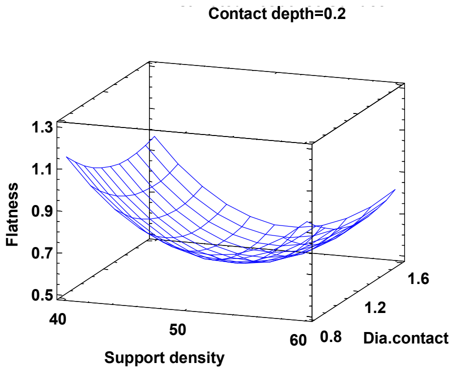

Figure S7. Estimated Response Surface for Flatness.

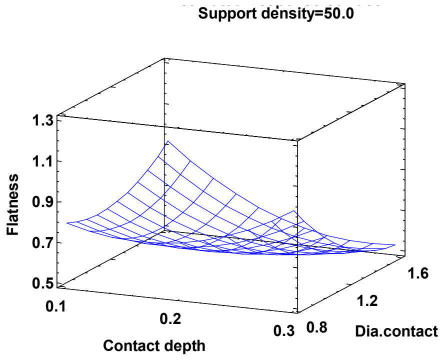

Figure S8. Estimated Response Surface for Flatness.

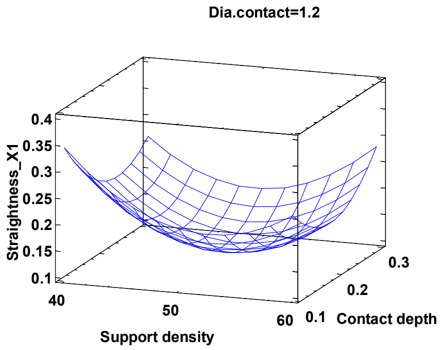

Figure S9. Estimated Response Surface for Straightness_X1.

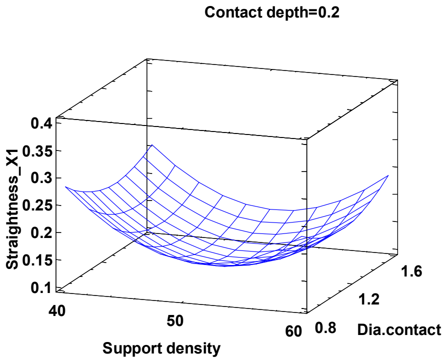

Figure S10. Estimated Response Surface for Straightness_X1.

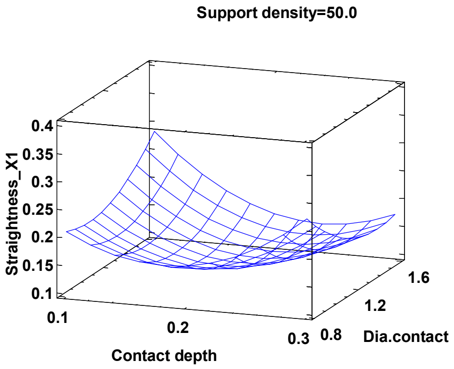

Figure S11. Estimated Response Surface for Straightness_X1.

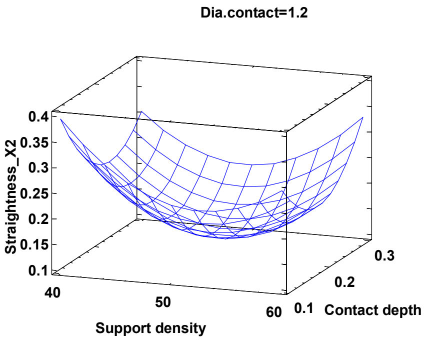

Figure S12. Estimated Response Surface for Straightness_X2.

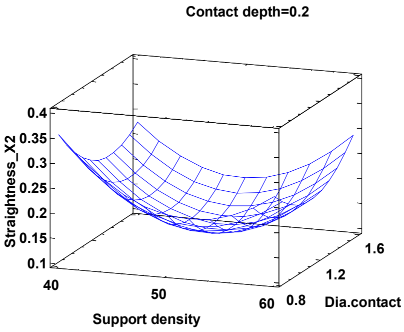

Figure S13. Estimated Response Surface for Straightness_X2.

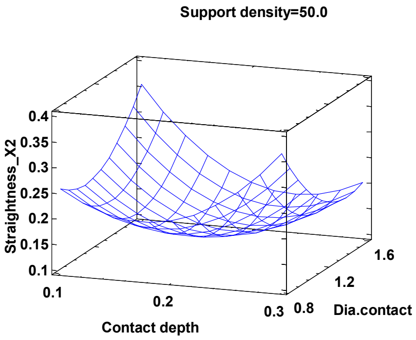

Figure S14. Estimated Response Surface for Straightness_X2.

9. The Estimated Response Surface for Straightness_Y1 and Straightness_Y2.

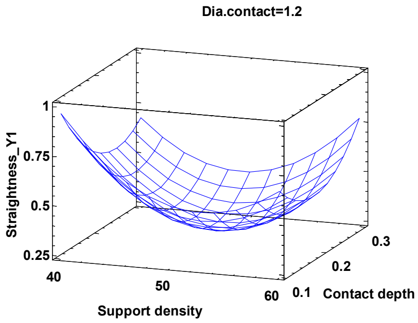

Figure S15. Estimated Response Surface for Straightness_Y1.

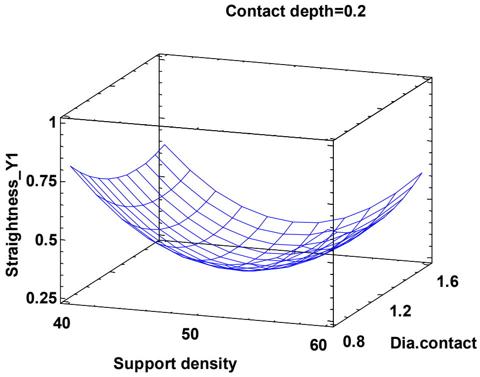

Figure S16. Estimated Response Surface for Straightness_Y1.

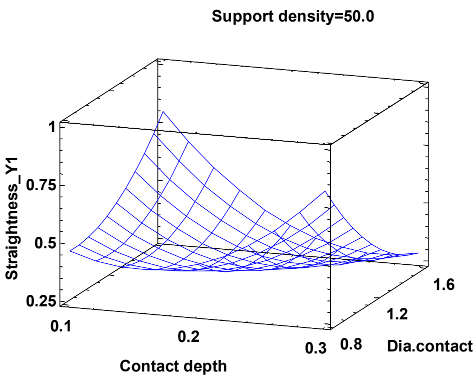

Figure S17. Estimated Response Surface for Straightness_Y1.

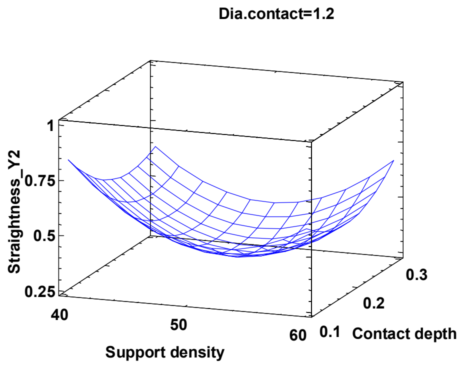

Figure S18. Estimated Response Surface for Straightness_Y2.

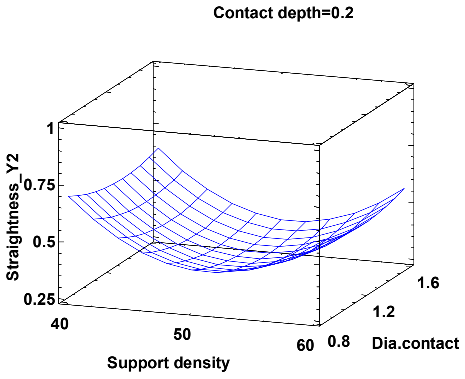

Figure S19. Estimated Response Surface for Straightness_Y2.

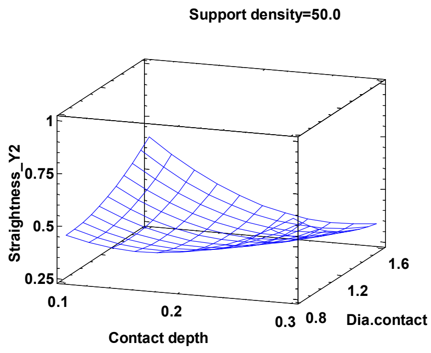

Figure S20. Estimated Response Surface for Straightness_Y2.

10. The Estimated Response Surface for Hole_15 and Circle_15_2.

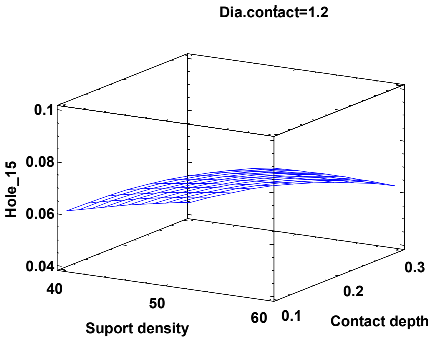

Figure S21. Estimated Response Surface for Roundness for Hole_15.

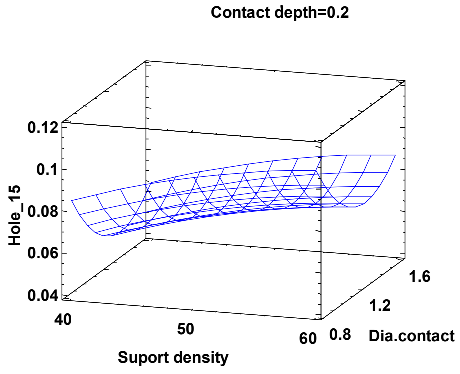

Figure S22. Estimated Response Surface for Roundness for Hole_15.

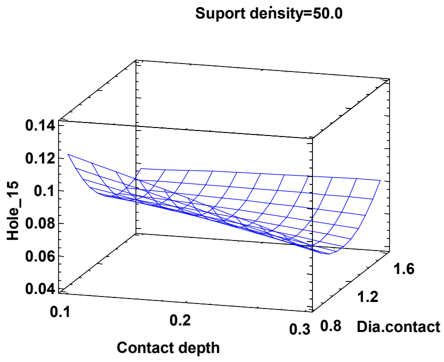

Figure S23. Estimated Response Surface for Roundness for Hole_15.

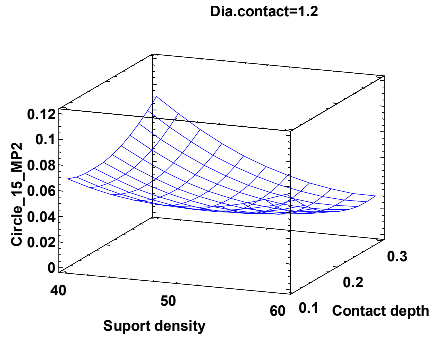

Figure S24. Estimated Response Surface for Roundness for Circle_15_2.

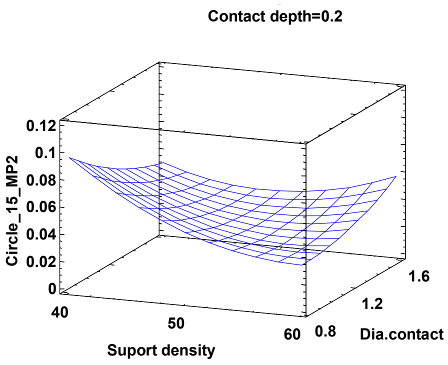

Figure S25. Estimated Response Surface for Roundness for Circle_15_2.

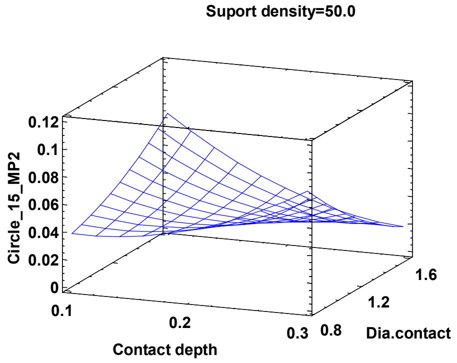

Figure S26. Estimated Response Surface for Roundness for Circle_15_2.

11. The experimental value for dimension supports– Straightness.

Table S7. The experimental value for dimension supports of the feature generated by 3D printing from solid design 3D element – Straightness.

Run	Independent factors			Response variables			
	Support density in %	Contact depth in mm	The diameter of the contact surface in mm	Straightness in mm			
				Along X-axis		Along Y-axis	
				Y1	Y2	X1	X2
1	40	0,1	1,2	0,862	0,826	0,337	0,330
2	40	0,2	1,6	0,679	0,668	0,260	0,304
3	40	0,3	1,2	0,686	0,673	0,258	0,302
4	40	0,2	0,8	0,871	0,685	0,287	0,394
5	50	0,2	1,2	0,310	0,349	0,122	0,136
6	50	0,1	1,6	0,871	0,658	0,287	0,394
7	50	0,1	0,8	0,499	0,502	0,209	0,280
8	50	0,3	0,8	0,751	0,630	0,267	0,326
9	50	0,2	1,2	0,311	0,348	***0,120***	***0,134***
10	50	0,2	1,2	0,309	0,350	0,124	0,135
11	50	0,3	1,6	***0,252***	***0,294***	0,173	0,180
12	60	0,3	1,2	0,871	0,685	0,287	0,394
13	60	0,2	1,6	0,558	0,602	0,231	0,250
14	60	0,2	0,8	0,598	0,571	0,223	0,242
15	60	0,1	1,2	0,659	0,589	0,256	0,293

12. The experimental value for dimension supports– Roundness.

Table S8. The experimental value for dimension supports of the feature generated by 3D printing from solid design 3D element – Roundness.

	**Independent factors**			**Response variables**					
**Run**	**Support density in %**	**Contact depth in mm**	**The diameter of the contact surface in mm**	**Roundness in mm**					
				**Hole**			**Cylinder**		
				Φ 5	Φ 10	Φ 15	Φ 5	Φ 10	Φ 15
							**Measuring plane**		
							MP 3	*MP 3*	*MP 3*
							MP 2	*MP 2*	*MP 2*
							MP 1	*MP 1*	*MP 1*
**(0)**	**(1)**	**(2)**	**(3)**	**(4)**	**(5)**	**(6)**	**(7)**	**(8)**	**(9)**
1	40	0,1	1,2	0,025	0,033	0,068	0,069	0.044	0,098
							0,045	0,189	0,059
							0,114	0,111	0,051
2	40	0,2	1,6	0,021	0,031	0,055	0,035	0,191	0,060
							0,064	0,149	0,043
							0,190	0,077	0,037
3	40	0,3	1,2	0,048	0,038	***0,045***	0,064	0,066	0,043
							0,066	0,088	0,092
							0,187	0,035	0,037
4	40	0,2	0,8	0,024	0,045	0,088	0,118	0,122	0,062
							0,131	0,049	0,113
							0,120	0,052	0,067
5	50	0,2	1,2	0,024	0,116	0,069	0,023	0,038	0,035
							0,021	0,051	0,041
							0,080	0,079	0,042
6	50	0,1	1,6	***0,018***	***0,043***	0,081	0,071	0,049	0,106
							0,059	0,037	0,102
							0,043	0,094	0,097
7	50	0,1	0,8	0,036	0,138	0,111	0,079	0,045	0,045
							0,032	0,047	0,030
							0,038	0,054	0,042
8	50	0,3	0,8	0,023	0,073	0,074	0,168	0,100	0,063
							0,024	0,041	0,068
							0,101	0,033	0,038
9	50	0,2	1,2	0,025	0,118	0,070	0,022	0,040	0,033
							0,022	0,050	0,042
							0,080	0,079	0,042
10	50	0,2	1,2	0,023	0,117	0,068	0,023	0,038	0,035
							0,021	0,051	0,041
							0,080	0,079	0,042
11	50	0,3	1,6	0,024	0,094	0,092	0,026	0,045	0,060
							0,022	0,056	0,027
							0,028	0,044	0,037
12	60	0,3	1,2	0,024	0,101	0,056	0,064	0,110	0,116
							0,051	0,040	0,040
							0,049	0,031	0,039
13	60	0,2	1,6	0,023	0,064	0,084	***0,030***	***0,034***	***0,081***
							0,027	***0,040***	***0,040***
							0,034	***0,054***	***0,048***

**(0)**	**(1)**	**(2)**	**(3)**	**(4)**	**(5)**	**(6)**	**(7)**	**(8)**	**(9)**
14	60	0,2	0,8	0,025	0,090	0,118	0,034	0,065	0,055
							0,037	0,058	0,041
							0,066	0,081	0,040
15	60	0,1	1,2	0,028	0,109	0,089	0,059	0,146	0.070
							0,049	0,043	0.073
							0,062	0,096	0,063
